# Type I Interferon Induction in Cutaneous DNA Damage Syndromes

**DOI:** 10.3389/fimmu.2021.715723

**Published:** 2021-07-23

**Authors:** Benjamin Klein, Claudia Günther

**Affiliations:** ^1^ Department of Dermatology, Venereology and Allergology, University Medicine Leipzig, Leipzig, Germany; ^2^ Department of Dermatology, University Hospital and Carl Gustav Carus, Technische Universität Dresden, Dresden, Germany

**Keywords:** Interferon, DNA damage, DNA repair, Werner syndrome (WS), Bloom Syndrome, Huriez syndrome, chilblain lupus, Ataxia teleangiectasia

## Abstract

Type I interferons (IFNs) as part of the innate immune system have an outstanding importance as antiviral defense cytokines that stimulate innate and adaptive immune responses. Upon sensing of pattern recognition particles (PRPs) such as nucleic acids, IFN secretion is activated and induces the expression of interferon stimulated genes (ISGs). Uncontrolled constitutive activation of the type I IFN system can lead to autoinflammation and autoimmunity, which is observed in autoimmune disorders such as systemic lupus erythematodes and in monogenic interferonopathies. They are caused by mutations in genes which are involved in sensing or metabolism of intracellular nucleic acids and DNA repair. Many authors described mechanisms of type I IFN secretion upon increased DNA damage, including the formation of micronuclei, cytosolic chromatin fragments and destabilization of DNA binding proteins. Hereditary cutaneous DNA damage syndromes, which are caused by mutations in proteins of the DNA repair, share laboratory and clinical features also seen in autoimmune disorders and interferonopathies; hence a potential role of DNA-damage-induced type I IFN secretion seems likely. Here, we aim to summarize possible mechanisms of IFN induction in cutaneous DNA damage syndromes with defects in the DNA double-strand repair and nucleotide excision repair. We review recent publications referring to Ataxia teleangiectasia, Bloom syndrome, Rothmund–Thomson syndrome, Werner syndrome, Huriez syndrome, and Xeroderma pigmentosum. Furthermore, we aim to discuss the role of type I IFN in cancer and these syndromes.

## Introduction

Type I interferons (IFNs), IFN *α* and IFN *β*, constitute a group of cytokines whose primary function is viral defense and protection against other intracellular pathogens (1). IFN secretion is activated after sensing of foreign- or self-nucleic acids (1). After binding on the interferon receptor (IFNAR), IFN is able to induce transcription of interferon stimulated genes (ISGs), resulting in activation of the innate immune system (1).

It has long been recognized that viral infections can induce flares of autoimmune diseases. This is mainly attributed to the upregulation of type I IFN which stimulates adaptive immunity and attenuates tolerance to self (2). Constitutive upregulation of type I IFN and ISG-transcription is seen in monogenic type I interferonopathies and autoinflammatory diseases such as systemic lupus erythematodes (2, 3). The importance of this group of cytokines is underlined by the prominent type I IFN signature found in blood of many complex autoimmune disorders such as dermatomyositis, systemic lupus erythematodes, Sjogren’s syndrome, and rheumatoid arthritis (4–8). Recent studies revealed type I IFN secretion after DNA damage through different mechanisms leading to distribution of nucleic components into the cytosol that will hereby be discussed (9–15).

DNA damage syndromes of the skin are caused by mutations in proteins taking part in the DNA repair (16–18). Due to UV-light driven carcinogenesis, these diseases often share the strong predisposition for the development of cutaneous malignancies, such as cutaneous squamous cell carcinoma (CSCC) and basal cell carcinoma (BCC) (16, 18). Important syndromes with defects in the DNA double-strand repair (DSBR) are Louis-Bar, Werner, Bloom, Rothmund–Thomson and Huriez syndromes (16, 18, 19). They can feature autoimmune phenotypes, such as positive antinuclear antibodies (ANA), rheumatoid arthritis, vitiligo, and scleratrophy. Here we summarize different mechanisms of IFN secretion, its role in DNA damage syndromes of the skin and discuss the role of the signaling pathway of type I IFN in cancer.

## Type I IFN—Activation and Secretion

**Figure 1:**
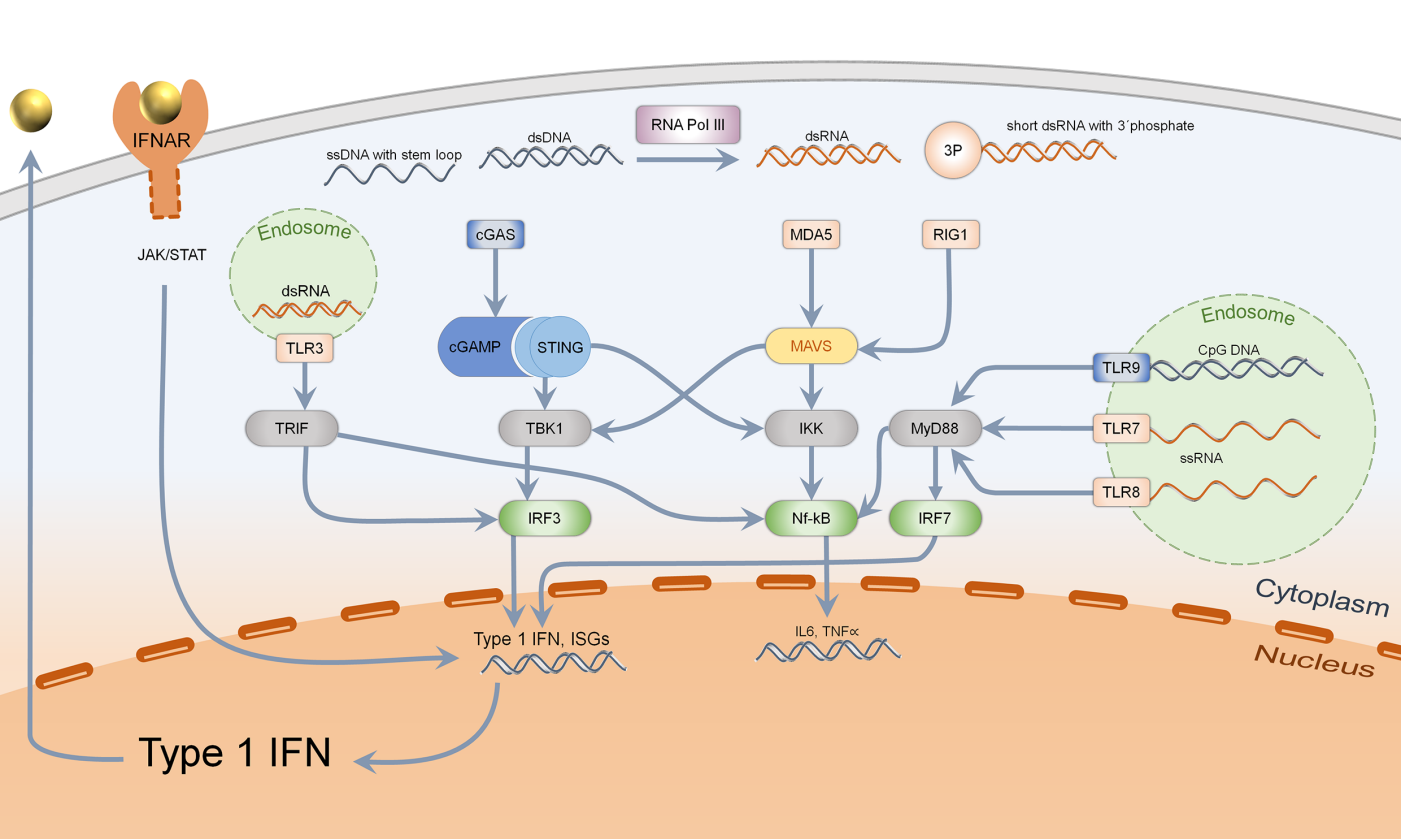
Sensing of intracellular nucleic acids and activation of type 1 IFN and Nf-kB pathway. Cytosolic DNA (marked in blue), specifically dsDNA and ssDNA with stem loop, is mainly detected by the cGAS–STING–TBK1–IRF3 pathway, leading to type 1 interferon secretion. Bacterial DNA with CpG motifs can furthermore be detected by endosomal TLR9 which acts *via* MyD88, IRF7, and Nf-kB. Cytosolic RNA is recognized by MDA5 (dsRNA) and RIG1 (short ds RNA with 3′phosphate), acting *via* MAVS to activate both TBK1-IRF3 and the Nf-kB pathway. Endosomal RNA is sensed by TLR3, 7, and 8 depending on the structure, activating either TRIF or MyD88. Activation of IRF3 and IRF7 leads to type 1 IFN transcription and secretion, which binds to IFNAR in an autocrine and paracrine manner.

Primary functions of type I IFN consist in the defense of bacterial and viral infections. Under steady state conditions, type I IFN is secreted upon sensing of pathogen associated molecular patterns (PAMPS) such as foreign nucleic acids (1). Nucleic acids can be detected by endosomal localized receptors such as TLRs (toll like receptors), which are mostly expressed by immune cells. TLR3 senses double-stranded RNA, TLR7, and TLR8 single-stranded RNA. TLR9 recognizes CpG (unmethylated cytosine-guanosine) DNA motifs which are typical for bacteria (20–22). TLRs activate adapter proteins, further inducing a signal cascade: TLR3 activates TRIF (TIR-domain-containing adapter inducing interferon ß), while TLR7, TLR8, and TLR9 stimulate MyD88 (myeloid differentiation primary response 88). TRIF induces activation of IRF3 (interferon regulatory factor 3), MyD88 acts *via* IRF7 (interferon regulatory factor 7) and both adapter proteins activate the NF-*κ*B-signaling pathway (20). Thereby, production of proinflammatory cytokines such as pro-IL1ß, pro-IL18, and secretion of type I IFN is enabled (**Figure 1**) (21).

Even in the cytosol, nucleic acids can be sensed by intracellular receptors which are expressed in almost every human cell. Short, double-stranded cytosolic RNA with a 5′triphosphate end is detected by RIG1 (retinoic acid inducible gene-I) while long, double-stranded RNA is recognized by MDA5 (melanoma differentiation associated gene 5) (1, 23, 24). They both activate MAVS (mitochondrial antiviral-signaling-protein) which acts through TBK1 (Tank-binding kinase 1), activating IRF3 (25). Cytosolic DNA can be detected by different mechanisms: On the one hand, the transformation of short, double-stranded RNA of AT-rich DNA *via* RNA Polymerase III acts through activation of RIG1/MAVS (26). On the other hand, single-stranded DNA with stem loop and double-stranded DNA are ligands of cGAS (cyclic GMP-AMP synthase), which generate cGAMP. This second messenger binds to the dimer STING (stimulator of interferon genes), which upon conformational change activates TBK1 and IRF3 (27). In addition, STING binds directly to bacterial cyclic dinucleotides (c-di-GMP and c-di-AMP), resulting in IRF3 activation (**Figure 1**) (28, 29). These different mechanisms only show an extract on how cytosolic nucleic acids can induce a type I IFN response.

Almost every human cell is capable of IFN *β* secretion, while IFN *α* is predominantly secreted by plasmacytoid dendritic cells (30, 31). Secreted type I IFN binds to IFNAR (interferon alpha-receptor) which consists of two heterodimers, IFNAR-1 and -2, which then activate the JAK/STAT pathway (januskinase/signal tranducers and activators of transcription) (32). A complex of STAT and IRF9 bind ISRE (interferon stimulated response element), resulting in transcription of ISGs (31). Subsequently, significant upregulated presentation of MHC-I, NK-cells and cytotoxic T-cells as well as proliferation of T-helper cells are activated (2). Moreover, autoreactive B-cell development and class switch from IgM to IgG are promoted which leads to autoantibody production (33). Type 1 IFN signaling thus promotes the development of a pro-inflammatory environment, which leads to autoimmunity due to a loss of tolerance in innate and adaptive immune responses.

The capacity of differentiation between self and foreign nucleic acids is limited. If the amount of self nucleic acids reaches a certain level in the cytosol, nucleic acid sensors can be activated, resulting in type 1 IFN secretion (34). To deteriorate the amount of nucleic acids, human cells exhibit cytosolic exonucleases such as TREX1 (three prime repair exonuclease 1), lysosomal DNase2, and extracellular DNase1 (35, 36). Deficiency of these proteins as well as gain of function mutations in nucleic acid sensors may lead to continuous stimulation of IFN secretion, which is observed in hereditary monogenic “interferonopathies”. These diseases are consistently featuring high levels of native ISG-expression, called “intrinsic interferone signature” (3). Loss of function mutations of TREX1, which were originally described in AGS (Aicardi Goutieres syndrome), result in accumulation of cytosolic DNA inducing a cGAS-dependent type I IFN secretion (37, 38). AGS patients show a broad phenotypic spectrum which is characterized by encephalopathy with dystonia, epilepsy, and microcephaly. Patients exhibit autoimmune symptoms such as Chilblain-Lupus, positive antinuclear antibodies and oral ulcerations (39, 40). Also, TREX1-associated familial Chilblain-Lupus is caused by a loss of function mutation in TREX1 which leads to activation of the type I IFN system. Patients show acral bluish red infiltrates and frequently develop systemic signs of lupus associated with a type I IFN signature in the blood (41–45).

Cutaneous DNA damage syndromes, characterized by defects in DNA repair proteins, can feature clinical phenotypes seen in autoimmune diseases such as autoantibody production, vitiligo, rheumatoid arthritis, and scleratrophy. In the following, the cutaneous DNA damage syndromes are presented in more detail, and the possible pathophysiological role of type 1 IFN in these diseases will be discussed.

## Cutaneous DNA Damage Syndromes With Defects in DNA Double-Strand Break Repair

Daily exposure to sunlight is the main inductor of skin aging, skin atrophy, and several malignancies such as cutaneous squamous cell carcinoma (CSCC) and basal cell carcinoma (BCC) (46). UVA and UVB exposition leads to several kinds of DNA damage *e.g.* UV light dose-dependent cyclopyrimidine-dimers (CPD) and oxidized bases (47, 48). Upon repair of this direct DNA damage, the formation of DNA DSB can be induced (48, 49). To repair these lesions, the DNA double-strand repair (DSBR) machinery is recruited which is divided into homologous recombination (HR) and non-homologous end joining (NHEJ), depending on the cell cycle.

Initiation of HR is the “DNA end resection”: Endonuclease activity of the MRE11-RAD50-NBS1-complex (Nijmegen breakage syndrome protein 1), DNA helicases (RECQ-helicase family), unwinding the helix structure, and exonucleases, cut out an ssDNA nucleotide to get a free 3′ end, where other repair enzymes of the DSBR can bind (50, 51). Afterwards, stabilizing proteins such as RAD51, RPA (replication protein A) and BRCA (BReast CAncer protein) are recruited to the free ssDNA (50). The homologous DNA strand of sister chromatid is used as template for “strand invasion”. Finally, the strands are reconnected (“strand annealing”) (50).

In NHEJ, DSBR is initiated by binding of Ku70/80 to the DSB, recruiting additional factors such as DNA-PKs (protein kinases) and XRCC4/DNA-ligase (50, 51). Further, nucleotide sequences in upstream and downstream of the DSB are excised by Artemis, DNA polymerase *λ* and *µ* (51). Then, DNA is ligated through DNA-ligase IV (50).

Defects in DNA DSBR are mostly located in defective DNA helicases (RECQ-helicase family). They usually affect the skin and several other tissues (**Table 1**) and often show autosomal recessive inheritance. Autosomal recessive Bloom syndrome is caused by heterozygous mutations in Bloom-helicase which is involved in DNA DSBR, HR (52, 61). The skin of patients shows telangiectasia and photosensitivity; furthermore patients have an elevated risk of developing leukemia, lymphoma, and gastrointestinal cancer and harbor defects in immune defense (17).

**Table 1:** Cutaneous double-strand break repair defects and symptoms (18, 52–60).

Disease	Skin symptoms	Extracutaneous symptoms and malignancy
*Affected protein*		
**Werner syndrome**	- progeroid phenotype (scleroatrophy) with ulcers - premature aging - alopecia - subcutaneous atrophy of the skin	- growth retardation - atherosclerosis with associated cardiovascular complications - cataract - diabetes mellitus type 2 - hypogonadism - malignancy (*e.g.* thyroid, melanoma, sarcoma)
***WRN helicase***		
**Rothmund–Thomson syndrome**	- photosensitivity - poikiloderma - alopecia - palmoplantar keratoderma - CSCC, BCC, melanoma	- juvenile cataract - saddle nose - osteosarcoma - hyposmia
***RECQL4 helicase***		
**Bloom syndrome**	- photosensitivity - telangiectasia - erythema (“butterfly” distribution - poikiloderma	- leukemia, lymphoma - gastrointestinal cancer - immunodeficiency
***BLM1 helicase***		
**Huriez syndrome**	- palmoplantar keratoderma - scleroatrophy of hands and feet - eczema - CSCC on lesional skin - telangiectasia - hypohidrosis	- not known
***SMARCAD1 (skin specific isoform)***		
**Ataxia teleangiectatica (Louis-Bar syndrome)**	- telangiectasia - vitiligo - premature hair graying	- rheumatoid arthritis - antinuclear antibodies - ataxia - immunodeficiency - malignancy (lymphoma, leukemia)
***ATM protein***		

Rothmund–Thomson syndrome represents an autosomal recessive disorder caused by homozygous and compound-heterozygous mutations of RECQL4-protein (62). Skin symptoms include poikiloderma (telangiectasia, change of pigmentation), hair loss, palmoplantar keratoderma, and patients may have a higher risk of developing BCC, CSCC, and melanoma. Patients often show abnormalities of the bones and can develop osteosarcomas (53, 54).

The progeroid Werner syndrome is characterized by premature aging upon defects in WRN helicase, which contains endonuclease activity (63). Clinical symptoms include skin atrophy, growth retardation, atherosclerosis, and high predisposition to different types of cancer (thyroid, melanoma, sarcoma) (17).

Recently discovered autosomal dominant Huriez syndrome (also known as sclerotylosis) is caused by mutations in the skin specific isoform of SMARCAD1 (SWI/SNF-related matrix-associated actin-dependent regulator of chromatin, subfamily A containing DEAD/H box 1), which is involved in DNA DSBR and chromatin remodeling (19, 64, 65). Patients exhibit palmoplantar keratoderma, palmoplantar scleroatrophy, onychodystrophy, adermatoglyphia, eczema, telangiectasia, and a high risk for developing CSCC at a young age (3rd to 4th decades) (55–57). Due to exclusive mutations in the skin specific isoform of SMARCAD1, which is mostly expressed in the skin and tongue, other malignancies of other cell types have not been described (19, 57, 58, 66).

## Cutaneous DNA Damage Syndromes With Defects in Nucleotide Excision Repair

Direct UV light-induced DNA damage such as CPDs and pyrimidine-pyrimidone (6–4) photoproducts is repaired by the nucleotide excision repair (NER) (67, 68). NER is divided in two mechanisms: transcription-coupled repair (TCR) and global genome repair (GCR). These pathways are different in recognition of “bulky lesions”: In TCR, RNA polymerase II, CSA, and CSB (Cockayne syndrome proteins A and B) recognize the damage, while in GGR XPC (xeroderma pigmentosum group C)/UV excision repair protein Rad23B or DDB1 and DDB2 (DNA damage binding proteins 1 and 2) are recruited (50, 68). Both pathways share the following steps: unwinding DNA (XPB, XPD) and dual excision of a 25–30 bp oligonucleotide by endonucleases at the 3′ side (XPG and TFIIH, transcription factor II H) and 5′ side (XPF and ERCC1 complex, excision repair cross complementation group 1) (50, 67, 68). The removed DNA is bound by RPA, while the gap is stabilized by RFC (replication factor C) and PCNA (proliferating cell nuclear antigen) and resynthesized by DNA polymerases (50, 67, 68). The two strands are annealed by DNA-ligase1/ligase III–XRCC1 complex (X-ray repair cross complementing protein 1) (50, 67, 68).

Diseases with defective proteins of NER include Xeroderma pigmentosum, Trichothiodystrophy (TTD), Cockayne syndrome (CS), and UV-sensitivity syndrome (UVSS) (**Table 2**) (16, 18). Exclusively, hereditary syndromes caused by mutations in proteins of GGR are associated with malignant transformation, while mutations in TCR are not (18). TTD, CS, and UVSS represent diseases with defects in TCR and will not be discussed in this review. Xeroderma pigmentosum is autosomal recessive caused by mutations in different genes (XPA-XPG) involved in NER (GGR), further enhancing malignant transformation (18, 69). Patients exhibit an extreme sensitivity to sunlight, show pigment changes and a highly elevated risk of developing skin cancer at a young age (69). The estimated increased risk is 10,000-fold higher for developing non-melanoma skin cancer and 2,000-fold higher for developing melanoma under the age of 20 (70). Ocular abnormalities are seen in UV-exposed structures of the eye. Of patients, 20–30% show neurological symptoms and intellectual deficiency (**Table 2**) (18, 69).

**Table 2:** Cutaneous nucleotide excision repair defects and symptoms (16, 18, 69, 70).

Disease	Skin symptoms	Extracutaneous symptoms and malignancy
*Affected protein*		
**Xeroderma pigmentosum group**	- extreme photosensitivity (acute photosensitivity in groups A, B, D, F, and G) - poikiloderma - freckling - premature photoaging - CSCC, BCC at a young age - melanoma at a young age	- ocular manifestations (*e.g.* keratitis) and ocular neoplasms - intraoral malignancies - neurological impairment (groups A, B, D and G, >50% of XPD) - intellectual impairment - lung cancer, leukemia, brain malignancies
***XPA***		
***XPB***		
***XPC***		
***XPD***		
***XPE***		
***XPF***		
***XPG***		
**Xeroderma pigmentosum variant**	- freckling - poikiloderma - premature photoaging - CSCC, BCC at a young age - melanoma at a young age	- ocular manifestations (*e.g.* keratitis) and ocular neoplasms - intraoral malignancies - intellectual impairment - lung cancer, leukemia, brain malignancies
***DNA Polymerase*** *η*		
**UV sensitivity syndrome**	- photosensitivity - pigment anomalies - telangiectasia - freckling	- no organ involvement
***ERCC8 (=CSA), ERCC6 (=CSB), UVSSA***		
**Trichothiodystrophy**	- photosensitivity - ichthyosis - brittle hair and nails	- intellectual impairment - decreased fertility - microcephaly - osteoporosis - cataract - hearing loss
***XPB***		
***XPD***		
***TTDA***		
**Cockayne syndrome**	- photosensitivity - anhidrosis - nail dystrophy	- microcephaly, stunted growth - progressive neurologic dysfunction - hearing loss
***ERCC8 (=CSA), ERCC6 (=CSB)***		

## Type I IFN Activation in DNA Damage Syndromes

Diseases associated with defects in DNA repair may also show autoimmune phenotypes: In Ataxia teleangiectatica, some patients show production of antinuclear antibodies (ANAs), symptomatic rheumatoid arthritis, and vitiligo besides the classic symptoms of ataxia, telangiectasia, and a high predisposition for the development of malignancies (10, 59, 60, 71, 72). It is caused by defects of the ATM protein, which is essential for a proper DNA double-strand repair (50). It has been proposed that autoimmune features are driven by a type I IFN response that is induced by byproducts of the DNA damage response. Härtlova et al. showed that cell stress is able to induce type I IFN system: Irradiated ATM-deficient cells show liberation of ssDNA-fragments which can penetrate into the cytosol and activate STING (10). To clarify if this mechanism is DNA damage-dependent, etoposide as specific inductor of DNA double-strand breaks showed significant activation of type I interferon. Moreover, lower concentrations of viral nucleic acids or c-di-GMP were necessary to induce a STING-dependent ISG expression in ATM-deficient cells. Authors claimed this phenomenon as “priming” of the type I IFN system (10).

Further, different studies of ATM deficiency led to the idea of mitochondrial DNA (mtDNA)-induced activation of cGAS and type I IFN (73–75): ATM, besides being detectable in the nucleus, is also detectable in mitochondrial fractions of human fibroblasts (74). ATM deficiency shows mitochondrial dysfunction which is associated with an innate immune response including type I IFN production (73). The mechanism was identified recently: ATM inhibition was shown to cause cytoplasmic leakage of mtDNA by downregulation of TFAM (mitochondrial transcription factor A), which is a mtDNA binding protein. Cytoplasmic mtDNA then activates cGAS-STING-dependent type I IFN secretion (75). Together, downregulation of TFAM caused by ATM deficiency promotes leakage of mtDNA into the cytosol and thus activates the type 1 IFN system. These results also show that ATM, in addition to its function in DNA double strand repair, is indirectly involved in the stabilization of mtDNA and mitochondrial homeostasis (75). Hence, different subcellular localizations and functions of ATM are responsible for type I IFN induction and may lead to the autoimmune phenotype observed in this disease.

The possible producer of DNA fragments during DNA repair, which further penetrate from the nucleus into the cytosol inducing a type I IFN response, was identified by Erdal et al.: Liberated ssDNA fragments are excised by Bloom-helicase (BLM) and Exonuclease 1 (Exo1) in the “DNA end resection” of HR as mentioned above: BLM1/Exo1-deficient cells exhibited significant lower expression of ISGs after irradiation compared to wild type, indicating less liberation of ssDNA into the cytosol (14).

**Figure 2:**
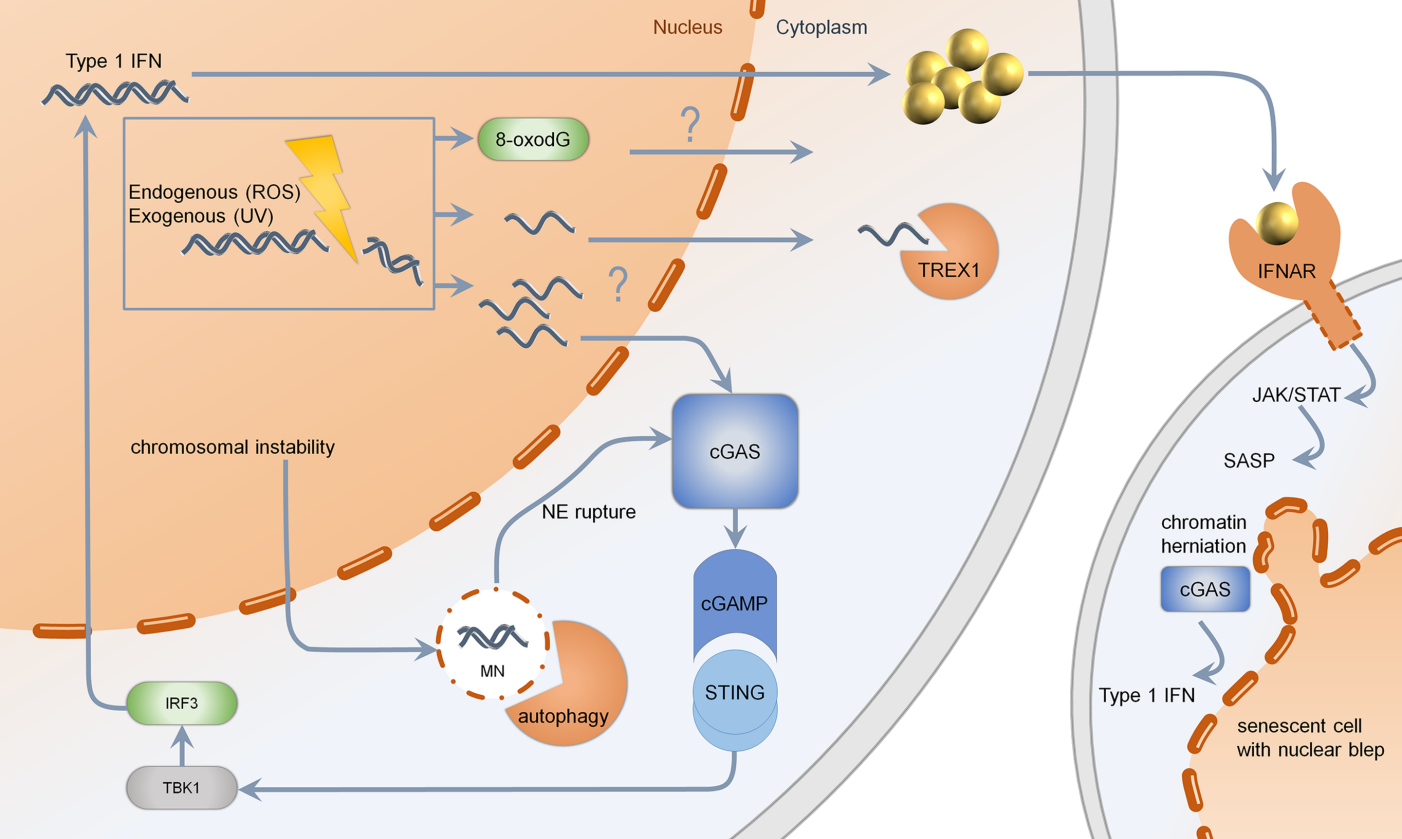
Mechanisms of type 1 IFN induction upon DNA damage. Genotoxic agents such as chemotherapeutics, UV-light, or reactive oxygen species (ROS) may lead to formation of ssDNA, which is normally cleared by TREX1. If TREX1 is deficient, the threshold for ssDNA is limited. Changes in DNA-bases such as 8-oxo-dG are resistant to enzymatic degradation *via* TREX1. Upon massive DNA damage or RAD51 deficiency, the generation of multiple cytosolic ssDNA fragments may lead to activation of cGAS. Cutaneous DNA damage syndromes, such as Bloom syndrome, characterized by chromosomal instability, show increased formation of micronuclei with fragile nuclear envelope. Normally, micronuclei are processed by autophagy. Upon rupture of the nuclear envelope, micronuclei are sensed by cGAS, resulting in production of cGAMP, further activating STING, TBK1, and IRF3. Secreted type 1 IFN binds to IFNAR, acting through the JAK–STAT pathway, leading to a senescent phenotype. Senescent cells, which are more frequent in cutaneous DNA damage syndromes, show nuclear blebs, also called chromatin herniations, which are able to activate the type 1 IFN system through cGAS. MN, micronucleus; NE, nuclear envelope; cGAS, cyclic GMP-AMP synthase; cGAMP, cyclic GMP-AMP; STING, stimulator of interferon genes; TBK1, Tank binding kinase 1; IRF, interferon regulatory factor; TREX1, three prime exonuclease 1; IFNAR, interferon alpha receptor; JAK, januskinase; STAT, signal transducers and activators of transcription; SASP, senescence-associated secretory phenotype; UV, ultraviolet; ROS, reactive oxygen species.

The transmission from nuclear DNA into the cytosol is protected by DNA binding proteins such as RPA and RAD51, which are upregulated upon DNA damage (13). Deficiency of RAD51 or associated proteins leads to liberation of ssDNA and dsDNA into the cytosol, further enhancing a type I IFN response in a STING-dependent manner (**Figure 2**) (13, 76).

Accumulated cytosolic ssDNA is normally cleaved by TREX1 (35). TREX1 mutations have been described to cause Aicardi Goutières syndrome and autosomal dominant familial chilblain lupus. Both diseases are characterized by spontaneous activation of the type I IFN system (37, 38, 41, 42). This is induced by ssDNA accumulating in the cytosol due to an activated DNA damage repair response (13, 77). TREX1 deficient cells are very competent in NER and DSB repair. They harbor replication stress and exhaustion of the RAD51 ssDNA-binding capacity facilitating DNA accumulation in the cytosol (13). The DNA sensor cGAS recognizes unrestricted DNA and stimulates STING (**Figure 2**) (27, 78). In TREX1-associated familial chilblain lupus, a deregulated IFN response was shown, which was enhanced by stimulators such as polyI:C (43). Recently, it has been shown that early type I IFN reactions upon UV-light in murine skin are cGAS dependent, suggesting a UV induced type I IFN dependent inflammation in cutaneous lupus erythematodes (79). Other mutations identified in Aicardi Goutières syndrome are defects in RNAseH2, which takes part in ribonucleotide excision repair and acts on RNA/DNA-hybrids, occurring during DNA replication (80, 81). Patients with defects in RNAseH2 accumulate ribonucleotides in DNA and show activation of the type I IFN system due to an activated DNA damage response. Lupus patients with defects in RNaseH2 are susceptible to UV-light due to enhanced CPD-formation in ribonucleotide-containing DNA (82). These results indicate that specific structural alterations of DNA are capable of type I IFN induction, further enhancing autoimmunity (**Figure 2**) (82).

Another way of releasing nucleic acids into the cytosol is the formation of genome instability-associated formation of micronuclei: a small nucleus with a lamin coated membrane. Due to instability of this membrane, DNA damage can lead to miscompartmentation, resulting in a cGAS-dependent activation of type I IFN (**Figure 2**) (11).

Werner syndrome patients exhibit scleroderma-like skin changes, which might be associated with an autoimmune phenotype. In Werner syndrome, the frequency of senescent cells is relatively high compared to normal controls (83). This is due to replicative senescence, which results from elevated telomer shortening (83, 84). Senescent cells show cell cycle arrest, resistance to growth factors and exhibit a higher amount of chromatin herniations compared to wild type (84, 85). Yu et al. showed significant activation of type I IFN in fibroblasts of patients with Werner syndrome (9). After treatment with anti-IFN antibodies they showed less cell cycle arrest, entrance in S-/G2M-phase of cell cycle, and a reduced rate of senescent cells. This indicates a potential disease-modifying role of type I IFN and possible therapeutic strategy: Antibodies against IFN could possibly stop premature aging and scleroderma skin changes in Werner syndrome (9). Interestingly, autoantibodies against WRN (Werner helicase) and a lower expression rate of WRN were observed in systemic sclerosis, suggesting a possible pathogenic link of sclerotic skin changes (15, 86). The WRN protein belongs to the RECQ-helicase family and contains an N-terminal 3′–5′ exonuclease activity. It represents a multifunctional nuclease involved in replication, telomer shortening, and DNA damage response maintaining genome integrity (87). It interacts with proteins in both NHEJ and HR (end resection) in DNA DSBR: In DSBR, it has an exonuclease function and is involved in “DNA end resection” (50). If type 1 IFN is activated by DNA fragments produced by end resection factors [according to Erdal et al. (14)], a deficiency of WRN would result in reduced excision of ssDNA, which represents a possible dangerous molecule in the cell. This alone cannot explain the increased type 1 IFN activation which was shown in patient cells and raises the question as to the source of type 1 IFN activation in Werner syndrome. The function of WRN protein is complex, and it has been shown that the non-enzymatic component of WRN protein is recruited by NBS1 to limit exonuclease activity of MRE11 at replication forks: In the absence of WRN, MRE11 degrades DNA during replication. NBS1 limits this process through recruitment of WRN (88). Further, WRN protein stabilizes RAD51, a DNA-binding protein (88). Together, WRN deficiency could lead to liberation of DNA of replication forks due to higher excision by MRE11 and destabilization of RAD51. RAD51 plays a pivotal role in maintaining DNA in the nucleus (13, 88). Deficiency of RAD51 in human fibroblast is sufficient to enhance IFN *β*-mRNA levels in the cell (13). Therefore, WRN deficiency and associated RAD51 destabilization could lead to accumulation of excised DNA, further activating IFN response. Further studies are needed to evaluate the impact of WRN deficiency, RAD51 destabilization and type I IFN.

Recent studies showed JAK-independent activation of STAT-signaling pathway in premature aging cells: The transcription factor ISGF3 (interferon stimulated gene factor 3), consisting of STAT1, STAT2, and IRF9 can induce ISG expression independently of IFN secretion in an unphosphorylated state: In aged cells and cells of patients with Werner syndrome, ISG expression was significantly upregulated compared to wild type (89, 90). This was caused by increased levels of unphosphorylated STAT1 and STAT2 proteins (89). JAK knockdown in these cells did not show a reduction of ISG expression (89). This effect may be due to post-translational modifications (*e.g.* acetylation, methylation) of STAT proteins in aging cells, activating ISGF3 (89, 90). Hence, JAK-independent activation of STAT proteins could explain enhanced ISG expression in this syndrome (89, 90).

Furthermore, the senescent phenotype in Werner syndrome, due to telomer shortening, could trigger the type I IFN system: chromatin herniations observed in senescence can be recognized by the immune sensor cGAS, inducing a STING-dependent type I IFN response (**Figure 2**) (12). Altogether, different mechanisms in Werner syndrome are able to induce the type I IFN system; hence more studies are needed to evaluate the precise substrate of type I IFN induction in Werner syndrome.

In contrast to the previous diseases, Bloom syndrome does not show a typical autoimmune phenotype; however, patients even show photosensitivity and symptoms of immunodeficiency such as more frequent respiratory and gastrointestinal infections (18, 91). Erdal et al. identified BLM helicase together with Exo1 as a possible source of ssDNA liberation of the nucleus into the cytosol upon DNA damage, inducing a IFN response (14). A deficient BLM protein, reducing ssDNA liberation, was associated with a diminished IFN response in breast cancer cells (14). However, Bloom syndrome represents a genetic instability syndrome, and elevated micronuclei formation was observed in patient cells (92). Gratia et al. showed a higher micronuclei formation and cGAS dependent induction of type I IFN in immortalized fibroblasts of Bloom syndrome patients (93). The frequency of cGAS colocalized micronuclei was not significantly altered, suggesting no upregulation of cGAS (93). Taken together, different pathomechanisms are possible in Bloom syndrome: Dependent on the amount of micronuclei and the activity of BLM1 helicase, IFN induction is possibly promoted or inhibited.

As to Rothmund Thomson syndrome, no recent studies have been published concerning DNA-damage-induced type I IFN, but significant higher rates of senescent cells have been described (54, 62). Patients exhibit photosensitivity but do not show autoimmune phenotypes such as the formation of antinuclear antibodies or scleroderma-like skin changes. As premature senescence is linked to chromatin herniation, chromatin mediated cGAS activation represents a possible mechanism of type I IFN induction. Furthermore, Rothmund Thomson syndrome as well as Werner syndrome was shown to be associated with mitochondrial dysfunction (94). As RECQL4 helicase plays a role in mtDNA replication and RECQL4 deficient cells exhibit higher mtDNA mutations (75, 95, 96), an mtDNA-driven type 1 IFN response similar to ATM deficiency seems possible (73, 75). However, it is not known, if Rothmund–Thomson syndrome is associated with an enhanced type I IFN activation.

In Huriez syndrome, caused by skin-specific SMARCAD1 deficiency, patients feature a scleroatrophic phenotype, also seen in systemic sclerosis, which marks a strongly associated type I IFN activation disease (97, 98). Recently, a link between the activation of type I IFN and higher DNA damage response in systemic sclerosis was shown, supporting the possible pathogenic role of DNA-damage-induced type I IFN in Huriez syndrome (15, 99). In systemic sclerosis, a higher rate of DNA damage was observed compared to wild type (15). Interestingly, higher IFN levels were associated with higher DNA damage burden, and accumulated DNA damage was proportional to the extent of fibrosis (15). The authors did not observe defects in the capacity of NER, but in DSBR (15). Mesenchymal stem cells of patients with systemic sclerosis reveal lower expression of SMARCAD1, suggesting Huriez syndrome and systemic sclerosis may share common pathways (100). Since SMARCAD1 represents a protein of the DSBR (HR), a similar mechanism seems possible. As part of DNA “end resection”, SMARCAD1, together with Exo1, excises ssDNA from DNA DSBs (64). This would be diminished in SMARCAD1 deficiency regarding similar functions of SMARCAD1 and BLM/Exo1 (14, 64). Hence, a lower IFN response compared to SMARCAD1 sufficiency would result. However, cells of patients with Huriez syndrome exhibit a high rate of senescent cells and show deficits in proliferation, yet it is unclear if chromatin herniation and an upregulated cGAS-induced IFN response are present in this syndrome (19). CSCC only occurs in lesional skin in these patients, potentially induced by chronic inflammation and reduced immunosurveillance which may be due to depletion of epidermal Langerhans cells (101, 102).

Xeroderma pigmentosum (XP) represents the most common hereditary cutaneous DNA damage syndrome and is caused by mutations in proteins of the nucleotide excision repair (XPA-XPG), as mentioned above (18, 46, 67). XP patients show cutaneous malignancies such as CSCC and BCC as well as melanoma in early childhood/puberty (18). Autoimmune phenotypes are not highly associated with the disease (18). Evidence of IFN induction in XP is very limited. Interestingly, a reduced native IFN response upon stimulation with polyI:C was observed in XP blood cells (103). However, a higher rate of micronuclei has been observed in XP group A fibroblasts representing a possible substrate of cGAS activation (104). It is not known if the amount of micronuclei formation is high enough to induce a relevant type I IFN response. IFN plays a pivotal role in immunosurveillance, leading to an antigen specific T-cell response against malignant cells (105, 106). Hence, a possible explanation for early malignancies in XP patients could be impaired immunosurveillance due to missing DNA-damage-induced type I IFN.

Interestingly, some of the cutaneous DNA damage syndromes with defects in DNA DSB show activation of the type I IFN system upon DNA damage induced by different mechanisms, reflecting clinical autoimmune phenotypes. In cutaneous DNA damage syndromes with defect NER, evidence for type I IFN induction is very limited or even not present. A possible explanation for type I IFN activation could be the damage itself: DSBs are highly mutagenic resulting in genome instability, enhancing the formation of micronuclei (11, 107, 108). Another reason could be the stronger DNA damage response in DSBR, leading to possible substrates for type I IFN induction (9, 109). Possible sources of DNA-damage-induced type I IFN in cutaneous DNA damage syndromes include genome instability-associated formation of micronuclei, leakage of mtDNA, activation of exonucleases upon high DNA damage, reduced capacity of nuclear DNA binding or cytosolic DNA degrading proteins as well as chromatin herniation in senescent cells which are highly associated with DNA damage syndromes (11–13, 35, 76, 82, 93).

## The Type I IFN System, Senescence, and Cancer

Activation of type I IFN raised importance in the context of cancer as an anti-tumorigenic mechanism of the cell, leading to immunosurveillance: Detection of tumor-derived DNA by innate immune sensors and cGAS-dependent activation of STING leads to type I IFN secretion (105, 106). Consequently, enhanced tumor antigen presentation and antigen-specific T-cell response as well as the recruitment of NK-cells are part of the anti-tumorigenic response (110). Type 1 IFN is required for anti-tumor response and tumor elimination in dendritic cells, and reduced IFN signaling was observed in different types of cancer such as colorectal carcinoma, melanoma, and pancreatic cancer (111, 112). Furthermore, IFNAR1 downregulation in cancer-associated stromal cells was observed in colon and pancreatic cancers (113). It was shown that suppression of STING is associated with less immune infiltration and subsequently increased tumor growth in melanoma (114). Downregulation of cGAS and STING was observed in clinically advanced tumors (115, 116), indicating a possible tumor-driven escape mechanism from immunosurveillance. Interestingly, Xeroderma pigmentosum shows the strongest association for the development of cutaneous malignancies and was associated with a deficiency in type 1 IFN production, which supports the idea of anti-tumorigenic effects of type 1 IFN signaling (103). As the other mentioned cutaneous DNA damage syndromes feature the formation of cancer despite certain activation of type 1 IFN, premature senescence and the chronic “senescence associated secretory phenotype” (SASP) might give an additional explanation of the clinical phenotype in these syndromes, which will be further discussed.

Senescence is a cellular phenomenon, characterized by cell cycle arrest and resistance to growth factors (84). The activation of the cGAS–STING pathway leads to secretion of inflammatory cytokines, chemokines, and proteases characterizing SASP (**Figure 2**) (117, 118). Chronic stress (such as DNA damage) and activation of SASP can be associated with induction of an immunosuppressive microenvironment leading to metastasis and resistance of DNA damaging agents such as chemotherapy (119). Thus, the SASP can paradoxically have both pro-tumorigenic and anti-tumorigenic functions: In one way, the recruitment of immune cells through IL6, CXCL1, and other cytokines mediate clearance of tumor cells (120). In another way, anti-inflammatory cytokines, such as IL10, secreted by senescent stromal cells, suppress anti-tumor immune responses. Hence, the cancer-inhibiting or cancer-promoting effect of SASP is tightly regulated and seems cell type- and cytokine-specific (120). The exact molecular mechanisms underlying SASP-induced tumor progression are not fully understood, but type I IFN in this context was shown to be downregulated by inhibition through p38, encoding for MAPK (mitogen activated protein kinase) (106). This downregulation of type I IFN could further explain the possible failure of immunosurveillance.

Taken together, the senescent phenotype in cutaneous DNA damage syndromes could be context-dependent: Acute activation of cGAS–STING enhances immunosurveillance *via* SASP, while the chronic secretion of inflammatory cytokines could have tumorigenic effects (105, 117, 118). It is not known if the observed premature senescence in cutaneous DNA damage syndromes is clearly pro- or anti-tumorigenic; this will be subject of future research. However, type I IFN induction represents an anti-tumorigenic mechanism (105, 111, 112), which may play a pivotal role in inhibiting skin cancer development in the cutaneous DNA damage syndromes.

## Therapeutic Implications

Skin changes in favor of scleroatrophy and premature aging as seen in progeroid syndromes, and Huriez syndrome may represent the effects of both chronic DNA damage and chronic inflammation, demonstrating the senescent phenotype (15, 98, 99). Therapeutic approaches against type I IFN (JAK inhibitors, anti-IFNAR antibodies, *etc.*) in cutaneous autoinflammatory and autoimmune diseases show promising effects in patients (44, 121, 122). To use these agents in type I IFN-driven inflammatory diseases seems plausible. However, it is not known if anti-IFN agents may possibly reduce immunosurveillance of evolving tumor cells in cutaneous DNA damage syndromes. IFNAR downregulation was observed in tumor progression of cancer associated stromal cells. Therefore, IFN reducing interventions need to be tested with caution to avoid enhanced tumor growth in cutaneous DNA damage syndromes.

## Perspectives

Molecular exploration of rare interferonopathies has improved our understanding of innate type I IFN driven immune responses and nucleic acid metabolism (3, 31, 123). It has further opened the view to DNA-damage-induced innate immune response and especially type 1 IFN induction. This exploration was mainly driven by description of ATM deficiency (10, 75). Although less is known in this regard for cutaneous DNA damage syndromes, the understanding of these rare diseases can help to elucidate molecular mechanism and to understand more complex diseases featuring similar clinical phenotypes. In Huriez syndrome, it will be interesting to explore under which conditions type 1 IFN might be upregulated (19). In Bloom, Werner, and Rothmund–Thomson syndromes, we still do not precisely know the molecular substrates that lead to type 1 IFN induction (9, 89, 93, 96). In this regard, it will be interesting to know if RAD51 destabilization is a possible mechanism of type 1 IFN induction in Werner syndrome. Finally, the physiological role of type 1 IFN for induction of senescence and its potential pro- or anti-tumorigenic effects warrant investigation.

## Author Contributions 

BK had full access to all of the data in the study and take responsibility for the integrity of the data and the accuracy of the data analysis. *Study concept and design*: BK and CG. *Acquisition, analysis, and interpretation of data*: BK and CG. *Drafting of the manuscript*: BK. *Critical revision of the manuscript for important intellectual content*: CG and BK. *Statistical analysis*: none. *Obtained funding*: CG. *Administrative, technical, or material support*: CG and BK. *Study supervision*: CG and BK. All authors contributed to the article and approved the submitted version.

## Funding

Funded by the Deutsche Forschungsgemeinschaft (DFG, German Research Foundation) – Project-ID 369799452 – TRR237.

## Conflict of Interest

The authors declare that the research was conducted in the absence of any commercial or financial relationships that could be construed as a potential conflict of interest.

## Publisher’s Note

All claims expressed in this article are solely those of the authors and do not necessarily represent those of their affiliated organizations, or those of the publisher, the editors and the reviewers. Any product that may be evaluated in this article, or claim that may be made by its manufacturer, is not guaranteed or endorsed by the publisher.
